# Triaqua­(1,10-phenanthroline-κ^2^ *N*,*N*′)(sulfato-κ*O*)zinc(II)

**DOI:** 10.1107/S1600536811003138

**Published:** 2011-01-29

**Authors:** Hong Liu, Hui Qin, Yun-Jie Zhang, Hong-Wei Yang, Jian Zhang

**Affiliations:** a College of Pharmacy, Jiamusi University, Jiamusi, Heilongjiang 154007, People’s Republic of China

## Abstract

The Zn(II) atom in the title compound, [Zn(SO_4_)(C_12_H_8_N_2_)(H_2_O)_3_], is coordinated by one O atom from a sulfate dianion, two N atoms from a 1,10-phenanthroline mol­ecule and three water O atoms in an octa­hedral geometry. An intra­molecular O—H⋯O hydrogen bond occurs. Inter­molecular O—H⋯O hydrogen bonds generate a layer structure parallel to (001). There are weak C—H⋯O inter­actions within the layers.

## Related literature

For related structures, see: An *et al.* (2007 ▶); Dietz *et al.* (2009 ▶); Harvey *et al.* (2000 ▶); Hu *et al.* (2009 ▶); Zheng *et al.* (2002 ▶).
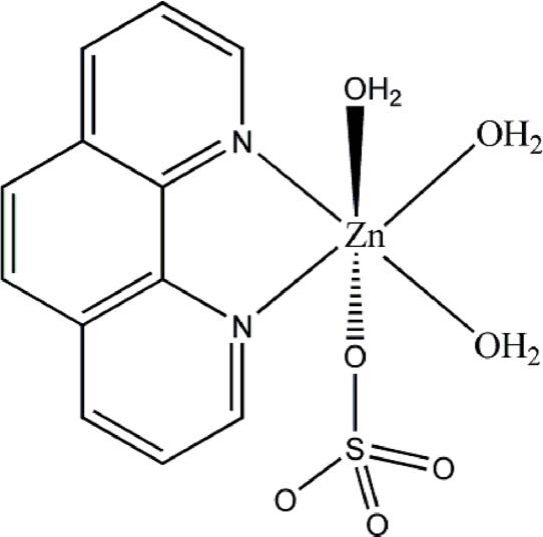


## Experimental

### 

#### Crystal data

[Zn(SO_4_)(C_12_H_8_N_2_)(H_2_O)_3_]
*M* *_r_* = 395.71
Orthorhombic, 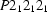

*a* = 8.0011 (4) Å
*b* = 9.6006 (4) Å
*c* = 19.1606 (9) Å
*V* = 1471.83 (12) Å^3^
*Z* = 4
Mo *K*α radiation
μ = 1.85 mm^−1^
*T* = 296 K
0.25 × 0.16 × 0.14 mm

#### Data collection

Bruker APEXII diffractometer
Absorption correction: multi-scan (*SADABS*; Sheldrick, 1996 ▶) *T*_min_ = 0.710, *T*_max_ = 0.785
7397 measured reflections
2793 independent reflections
2667 reflections with *I* > 2σ(*I*)
*R*_int_ = 0.025

#### Refinement

*R*[*F* ^2^ > 2σ(*F* ^2^)] = 0.021
*wR*(*F* ^2^) = 0.050
*S* = 1.01
2793 reflections
208 parameters
H-atom parameters constrained
Δρ_max_ = 0.29 e Å^−3^
Δρ_min_ = −0.36 e Å^−3^
Absolute structure: Flack (1983 ▶), 1165 Friedel pairs
Flack parameter: 0.005 (9)

### 

Data collection: *APEX2* (Bruker, 2003 ▶); cell refinement: *SAINT* (Bruker, 2001 ▶); data reduction: *SAINT*; program(s) used to solve structure: *SHELXS97* (Sheldrick, 2008 ▶); program(s) used to refine structure: *SHELXL97* (Sheldrick, 2008 ▶); molecular graphics: *DIAMOND* (Brandenburg & Berndt, 1999 ▶); software used to prepare material for publication: *SHELXL97*.

## Supplementary Material

Crystal structure: contains datablocks I, global. DOI: 10.1107/S1600536811003138/ng5108sup1.cif

Structure factors: contains datablocks I. DOI: 10.1107/S1600536811003138/ng5108Isup2.hkl

Additional supplementary materials:  crystallographic information; 3D view; checkCIF report

## Figures and Tables

**Table 1:** Selected bond lengths (Å)

Zn—O7	2.0874 (16)
Zn—O5	2.1128 (15)
Zn—O6	2.1175 (16)
Zn—O1	2.1431 (15)
Zn—N2	2.1442 (19)
Zn—N1	2.1605 (19)

**Table 2:** Hydrogen-bond geometry (Å, °)

*D*—H⋯*A*	*D*—H	H⋯*A*	*D*⋯*A*	*D*—H⋯*A*
O5—H51⋯O2^i^	0.87	1.79	2.655 (2)	174
O5—H52⋯O4^ii^	0.87	1.91	2.775 (2)	172
O6—H61⋯O1^ii^	0.91	1.93	2.809 (2)	161
O6—H62⋯O3	0.87	1.86	2.688 (2)	158
O7—H71⋯O3^iii^	0.96	1.85	2.769 (3)	160
O7—H72⋯O4^i^	0.83	1.97	2.797 (2)	173
C1—H1⋯O3^iii^	0.93	2.51	3.416 (3)	165

Symmetry codes: (i) 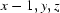; (ii) 
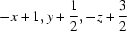
; (iii) 
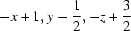
.

**Fig. 1.**
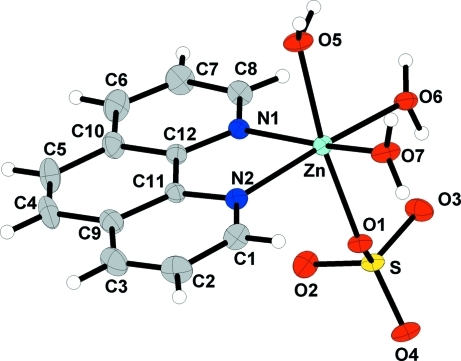
The coordiantion feature of the title compound. Dispalcement ellipsoids are drawn at the 30% probability level.

**Fig. 2.**
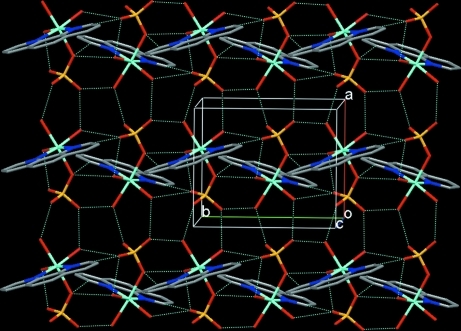
A view of two-dimensional layered structures of the title compound (color codes: Zn, cyan; S, yellow; O, red; N, blue; C, grey.). H atoms are omitted for clarity.

## supplementary crystallographic information

### Comment

The design and synthesis of 1,10-phenanthroline (phen) and sulfato coordination compounds have drawn considerable attention in the last decade. For the 0D structures of phen and sulfato ligand based transition-metal complexes, see: Zheng *et al.* (2002); An *et al*. (2007); Harvey *et al.* (2000). For the chian structures, see: Dietz *et al.* (2009); Hu *et al.* (2009). Among these compounds, the zinc(II) complexes were reported by Harvey *et al.* (2000) and Hu *et al.* (2009). We herein report a new phen and sulfato coordinated zinc(II) complex, which presents similar coordiantion style for zinc atom of that reported by Harvey *et al.* (2000).

The title compound crystallizes in chiral *P*2_1_2_1_2_1_ space group with three twofold axizes coexisting along *a*, *b* and *c* axies respectively, which is firstly found among the known phen and sulfato ligand based transition-metal complexes. As shown in Fig. 1, the Zn atoms are each surrounded by two N atoms from one phen ligand, four O atoms from three water molecules and one sulfato group to form distorted octahedra. The distances of Zn—N are 2.1591 (19) Å and 2.1449 (19) Å, respectively. The distances of Zn—O are in the range of 2.0881 (17) Å to 2.1443 (15) Å. The complex molecule displays a strong intramolecular hydrogen bond between water O(6) and sulfato O(3) atoms with *d*(O6···O3) = 2.687 Å. The intermolecular O—H···O hydrogen bonds exist between water molecules and sulfato O atoms and favor the formation of two-dimensional layered supramolecular network along [0 0 1] direction (Fig. 2). There are also weak C—H···O interactions between phen and sulfato O atoms to consolidate the two-dimensional framework. Different to the known phen and sulfato ligand based complexes, the neighboring phen ligands in the title compound do not exist the transparent π—π stacking interactions. This result indicates that solventothermal synthetic procedure may restrain the formation of π—π stacking interactions between neighboring conjugated ligands.

### Experimental

A mixture of ZnSO_4_ (0.0719 g, 0.25 mmol) and 1,10-phenanthroline (0.0496 g, 0.25 mmol) was dissolved in 8 ml 60% (*V*:*V*) ethanol solution and stirred for about an hour. Then it was transferred and sealed in a 15 ml Teflon-lined bomb which was heated at 100 °C for 5 days and cooled to room temperature. Colorless block crystals were collected. Yield: *ca*. 25% based on Zn. Elementalanalysis (%): Calcd for C_12_H_14_N_2_O_7_SZn: C, 36.42; H, 3.57; N, 7.08; Zn, 16.53. Found: C, 36.04; H, 3.68; N, 7.02; Zn, 16.39.

### Refinement

The disagreeable reflections 0 4 1 and 2 0 0 have been omitted and there are 1116 Friedel pairs in the refinement. H atoms on phen ligand were added theoreticlly with C—H distance of 0.93 Å. H atoms of water molecules were located from difference Fourier maps and then fixed to O atoms. All H atoms were allocated displacement parameters related to those of their parent atoms [*U*_iso_(H) = 1.2 *U*_eq_ (C or O)].

### Crystal data

**Table:**

[Zn(SO_4_)(C_12_H_8_N_2_)(H_2_O)_3_]	*F*(000) = 808
*M**_r_* = 395.71	*D*_x_ = 1.786 Mg m^−^^3^
Orthorhombic, *P*2_1_2_1_2_1_	Mo *K*α radiation, λ = 0.71073 Å
Hall symbol: P 2ac 2ab	Cell parameters from 1025 reflections
*a* = 8.0011 (4) Å	θ = 1.5–26.5°
*b* = 9.6006 (4) Å	µ = 1.85 mm^−^^1^
*c* = 19.1606 (9) Å	*T* = 296 K
*V* = 1471.83 (12) Å^3^	Block, colourless
*Z* = 4	0.25 × 0.16 × 0.14 mm

### Data collection

**Table:**

Bruker APEXII diffractometer	2793 independent reflections
Radiation source: fine-focus sealed tube	2667 reflections with *I* > 2σ(*I*)
graphite	*R*_int_ = 0.025
ω and φ scans	θ_max_ = 26.0°, θ_min_ = 2.1°
Absorption correction: multi-scan (*SADABS*; Sheldrick, 1996)	*h* = −9→9
*T*_min_ = 0.710, *T*_max_ = 0.785	*k* = −11→11
7397 measured reflections	*l* = −22→23

### Refinement

**Table:**

Refinement on *F*^2^	Secondary atom site location: difference Fourier map
Least-squares matrix: full	Hydrogen site location: inferred from neighbouring sites
*R*[*F*^2^ > 2σ(*F*^2^)] = 0.021	H-atom parameters constrained
*wR*(*F*^2^) = 0.050	*w* = 1/[σ^2^(*F*_o_^2^) + (0.0161*P*)^2^ + 0.005*P*] where *P* = (*F*_o_^2^ + 2*F*_c_^2^)/3
*S* = 1.01	(Δ/σ)_max_ = 0.001
2793 reflections	Δρ_max_ = 0.29 e Å^−^^3^
208 parameters	Δρ_min_ = −0.36 e Å^−^^3^
0 restraints	Absolute structure: Flack (1983), 1165 Friedel pairs
Primary atom site location: structure-invariant direct methods	Flack parameter: 0.005 (9)

### Special details

**Table:**

Geometry. All e.s.d.'s (except the e.s.d. in the dihedral angle between two l.s. planes) are estimated using the full covariance matrix. The cell e.s.d.'s are taken into account individually in the estimation of e.s.d.'s in distances, angles and torsion angles; correlations between e.s.d.'s in cell parameters are only used when they are defined by crystal symmetry. An approximate (isotropic) treatment of cell e.s.d.'s is used for estimating e.s.d.'s involving l.s. planes.
Refinement. Refinement of *F*^2^ against ALL reflections. The weighted *R*-factor *wR* and goodness of fit *S* are based on *F*^2^, conventional *R*-factors *R* are based on *F*, with *F* set to zero for negative *F*^2^. The threshold expression of *F*^2^ > σ(*F*^2^) is used only for calculating *R*-factors(gt) *etc*. and is not relevant to the choice of reflections for refinement. *R*-factors based on *F*^2^ are statistically about twice as large as those based on *F*, and *R*- factors based on ALL data will be even larger.

### Fractional atomic coordinates and isotropic or equivalent isotropic displacement parameters (Å^2^)

**Table:**

	*x*	*y*	*z*	*U*_iso_*/*U*_eq_	
Zn	0.35962 (3)	0.46528 (2)	0.682343 (13)	0.02381 (7)	
S	0.75438 (7)	0.43014 (5)	0.72471 (3)	0.02418 (13)	
O1	0.59078 (18)	0.36372 (14)	0.70689 (8)	0.0256 (4)	
O2	0.8188 (2)	0.50361 (18)	0.66363 (10)	0.0400 (5)	
O3	0.7288 (2)	0.52839 (19)	0.78257 (9)	0.0405 (4)	
O4	0.8701 (2)	0.31908 (16)	0.74536 (10)	0.0368 (4)	
O5	0.13662 (19)	0.58142 (15)	0.67227 (9)	0.0360 (4)	
H51	0.0345	0.5516	0.6719	0.043*	
H52	0.1259	0.6578	0.6961	0.043*	
O6	0.3974 (2)	0.57293 (15)	0.77750 (9)	0.0323 (4)	
H61	0.3775	0.6643	0.7869	0.039*	
H62	0.5003	0.5579	0.7908	0.039*	
O7	0.2180 (2)	0.31186 (16)	0.73196 (10)	0.0371 (4)	
H71	0.2385	0.2140	0.7387	0.044*	
H72	0.1156	0.3184	0.7392	0.044*	
N1	0.4592 (2)	0.62126 (19)	0.61251 (10)	0.0264 (4)	
N2	0.3537 (3)	0.35890 (18)	0.58398 (10)	0.0291 (4)	
C1	0.3124 (3)	0.2282 (3)	0.57089 (15)	0.0384 (6)	
H1	0.2798	0.1720	0.6080	0.046*	
C2	0.3153 (4)	0.1707 (3)	0.50423 (16)	0.0489 (8)	
H2	0.2882	0.0775	0.4975	0.059*	
C3	0.3584 (4)	0.2526 (3)	0.44882 (16)	0.0491 (7)	
H3	0.3586	0.2159	0.4039	0.059*	
C4	0.4539 (4)	0.4834 (4)	0.40547 (14)	0.0534 (8)	
H4	0.4550	0.4519	0.3596	0.064*	
C5	0.5013 (4)	0.6158 (4)	0.41983 (16)	0.0560 (8)	
H5	0.5333	0.6740	0.3834	0.067*	
C6	0.5501 (4)	0.8055 (3)	0.50634 (16)	0.0513 (8)	
H6	0.5799	0.8678	0.4713	0.062*	
C7	0.5510 (4)	0.8461 (3)	0.57495 (17)	0.0478 (7)	
H7	0.5808	0.9366	0.5870	0.057*	
C8	0.5069 (3)	0.7504 (3)	0.62626 (15)	0.0356 (6)	
H8	0.5112	0.7786	0.6727	0.043*	
C9	0.4024 (3)	0.3920 (3)	0.45999 (14)	0.0385 (6)	
C10	0.5032 (4)	0.6680 (3)	0.48954 (14)	0.0401 (6)	
C11	0.4028 (3)	0.4397 (2)	0.52967 (12)	0.0270 (5)	
C12	0.4559 (3)	0.5801 (2)	0.54475 (13)	0.0274 (5)	

### Atomic displacement parameters (Å^2^)

**Table:**

	*U*^11^	*U*^22^	*U*^33^	*U*^12^	*U*^13^	*U*^23^
Zn	0.02190 (13)	0.02351 (11)	0.02601 (13)	−0.00085 (10)	−0.00003 (11)	0.00258 (11)
S	0.0178 (3)	0.0195 (2)	0.0353 (3)	−0.00060 (19)	−0.0031 (2)	−0.0023 (2)
O1	0.0160 (7)	0.0222 (7)	0.0387 (10)	−0.0020 (6)	−0.0036 (7)	−0.0028 (6)
O2	0.0267 (9)	0.0472 (11)	0.0462 (11)	−0.0069 (7)	0.0022 (7)	0.0116 (8)
O3	0.0367 (9)	0.0395 (9)	0.0453 (11)	0.0022 (9)	−0.0069 (8)	−0.0196 (9)
O4	0.0234 (8)	0.0299 (8)	0.0570 (11)	0.0054 (7)	−0.0058 (9)	0.0058 (7)
O5	0.0207 (8)	0.0286 (7)	0.0589 (11)	0.0011 (7)	−0.0020 (9)	0.0013 (8)
O6	0.0331 (9)	0.0277 (8)	0.0361 (9)	0.0088 (6)	−0.0016 (7)	−0.0025 (7)
O7	0.0239 (9)	0.0271 (8)	0.0601 (12)	−0.0002 (7)	0.0102 (8)	0.0125 (8)
N1	0.0217 (10)	0.0288 (9)	0.0288 (11)	−0.0013 (8)	0.0015 (8)	0.0004 (8)
N2	0.0267 (10)	0.0287 (8)	0.0317 (10)	−0.0010 (9)	−0.0033 (10)	−0.0012 (8)
C1	0.0392 (16)	0.0324 (12)	0.0436 (16)	−0.0071 (11)	−0.0047 (12)	0.0014 (11)
C2	0.0497 (18)	0.0393 (14)	0.0578 (19)	−0.0062 (13)	−0.0099 (14)	−0.0176 (13)
C3	0.0487 (17)	0.0583 (16)	0.0402 (16)	−0.0022 (15)	−0.0058 (15)	−0.0209 (14)
C4	0.0595 (18)	0.076 (2)	0.0244 (14)	−0.0044 (17)	0.0069 (12)	−0.0032 (14)
C5	0.068 (2)	0.069 (2)	0.0307 (16)	−0.0089 (17)	0.0116 (15)	0.0113 (15)
C6	0.0566 (19)	0.0457 (16)	0.0515 (19)	−0.0135 (14)	0.0086 (15)	0.0187 (14)
C7	0.0550 (18)	0.0332 (13)	0.0553 (19)	−0.0153 (13)	0.0078 (15)	0.0037 (13)
C8	0.0347 (14)	0.0338 (12)	0.0383 (15)	−0.0065 (11)	0.0018 (12)	−0.0044 (11)
C9	0.0332 (15)	0.0476 (14)	0.0347 (14)	0.0019 (11)	−0.0005 (11)	−0.0067 (12)
C10	0.0385 (15)	0.0482 (15)	0.0338 (15)	−0.0037 (13)	0.0085 (12)	0.0071 (12)
C11	0.0206 (11)	0.0342 (12)	0.0262 (12)	0.0019 (9)	0.0003 (9)	−0.0021 (9)
C12	0.0221 (12)	0.0324 (11)	0.0277 (12)	0.0007 (9)	0.0014 (10)	0.0009 (10)

### Geometric parameters (Å, °)

**Table:**

Zn—O7	2.0874 (16)	C1—C2	1.392 (4)
Zn—O5	2.1128 (15)	C1—H1	0.9300
Zn—O6	2.1175 (16)	C2—C3	1.365 (4)
Zn—O1	2.1431 (15)	C2—H2	0.9300
Zn—N2	2.1442 (19)	C3—C9	1.400 (4)
Zn—N1	2.1605 (19)	C3—H3	0.9300
S—O2	1.4605 (18)	C4—C5	1.355 (5)
S—O4	1.4663 (16)	C4—C9	1.425 (4)
S—O3	1.4698 (18)	C4—H4	0.9300
S—O1	1.4955 (15)	C5—C10	1.427 (4)
O5—H51	0.8655	C5—H5	0.9300
O5—H52	0.8678	C6—C7	1.371 (4)
O6—H61	0.9094	C6—C10	1.409 (4)
O6—H62	0.8738	C6—H6	0.9300
O7—H71	0.9628	C7—C8	1.392 (4)
O7—H72	0.8331	C7—H7	0.9300
N1—C8	1.324 (3)	C8—H8	0.9300
N1—C12	1.357 (3)	C9—C11	1.412 (3)
N2—C1	1.322 (3)	C10—C12	1.405 (3)
N2—C11	1.356 (3)	C11—C12	1.442 (3)
			
O7—Zn—O5	87.46 (6)	N2—C1—C2	123.1 (3)
O7—Zn—O6	91.71 (7)	N2—C1—H1	118.4
O5—Zn—O6	86.66 (6)	C2—C1—H1	118.4
O7—Zn—O1	92.72 (6)	C3—C2—C1	119.3 (2)
O5—Zn—O1	171.49 (6)	C3—C2—H2	120.3
O6—Zn—O1	84.83 (6)	C1—C2—H2	120.3
O7—Zn—N2	92.99 (7)	C2—C3—C9	119.7 (3)
O5—Zn—N2	98.77 (7)	C2—C3—H3	120.2
O6—Zn—N2	172.97 (7)	C9—C3—H3	120.2
O1—Zn—N2	89.73 (7)	C5—C4—C9	120.7 (3)
O7—Zn—N1	166.26 (7)	C5—C4—H4	119.7
O5—Zn—N1	83.64 (6)	C9—C4—H4	119.7
O6—Zn—N1	98.18 (7)	C4—C5—C10	121.5 (3)
O1—Zn—N1	97.64 (6)	C4—C5—H5	119.2
N2—Zn—N1	78.11 (7)	C10—C5—H5	119.2
O2—S—O4	110.15 (10)	C7—C6—C10	119.2 (3)
O2—S—O3	110.09 (11)	C7—C6—H6	120.4
O4—S—O3	110.55 (11)	C10—C6—H6	120.4
O2—S—O1	109.39 (10)	C6—C7—C8	119.2 (3)
O4—S—O1	107.69 (9)	C6—C7—H7	120.4
O3—S—O1	108.91 (10)	C8—C7—H7	120.4
S—O1—Zn	127.69 (8)	N1—C8—C7	123.5 (3)
Zn—O5—H51	128.6	N1—C8—H8	118.3
Zn—O5—H52	118.8	C7—C8—H8	118.3
H51—O5—H52	101.0	C3—C9—C11	117.1 (2)
Zn—O6—H61	128.0	C3—C9—C4	123.3 (3)
Zn—O6—H62	107.7	C11—C9—C4	119.5 (2)
H61—O6—H62	105.5	C12—C10—C6	117.5 (2)
Zn—O7—H71	131.1	C12—C10—C5	119.4 (3)
Zn—O7—H72	123.8	C6—C10—C5	123.1 (3)
H71—O7—H72	102.6	N2—C11—C9	122.6 (2)
C8—N1—C12	118.0 (2)	N2—C11—C12	117.8 (2)
C8—N1—Zn	129.22 (18)	C9—C11—C12	119.6 (2)
C12—N1—Zn	112.53 (14)	N1—C12—C10	122.7 (2)
C1—N2—C11	118.0 (2)	N1—C12—C11	118.0 (2)
C1—N2—Zn	128.65 (18)	C10—C12—C11	119.3 (2)
C11—N2—Zn	113.30 (14)		
			
O2—S—O1—Zn	−66.81 (14)	C12—N1—C8—C7	1.1 (4)
O4—S—O1—Zn	173.47 (11)	Zn—N1—C8—C7	−172.2 (2)
O3—S—O1—Zn	53.56 (15)	C6—C7—C8—N1	−1.8 (5)
O7—Zn—O1—S	−144.44 (12)	C2—C3—C9—C11	1.3 (4)
O6—Zn—O1—S	−52.97 (12)	C2—C3—C9—C4	178.0 (3)
N2—Zn—O1—S	122.58 (13)	C5—C4—C9—C3	−177.4 (3)
N1—Zn—O1—S	44.62 (13)	C5—C4—C9—C11	−0.8 (4)
O7—Zn—N1—C8	127.7 (3)	C7—C6—C10—C12	1.4 (4)
O5—Zn—N1—C8	77.8 (2)	C7—C6—C10—C5	−178.9 (3)
O6—Zn—N1—C8	−7.9 (2)	C4—C5—C10—C12	0.4 (5)
O1—Zn—N1—C8	−93.7 (2)	C4—C5—C10—C6	−179.3 (3)
N2—Zn—N1—C8	178.2 (2)	C1—N2—C11—C9	3.6 (3)
O7—Zn—N1—C12	−45.9 (4)	Zn—N2—C11—C9	−177.59 (18)
O5—Zn—N1—C12	−95.77 (15)	C1—N2—C11—C12	−176.9 (2)
O6—Zn—N1—C12	178.55 (15)	Zn—N2—C11—C12	1.9 (3)
O1—Zn—N1—C12	92.71 (15)	C3—C9—C11—N2	−3.9 (4)
N2—Zn—N1—C12	4.61 (15)	C4—C9—C11—N2	179.3 (2)
O7—Zn—N2—C1	−15.4 (2)	C3—C9—C11—C12	176.6 (2)
O5—Zn—N2—C1	−103.3 (2)	C4—C9—C11—C12	−0.2 (4)
O1—Zn—N2—C1	77.3 (2)	C8—N1—C12—C10	1.0 (3)
N1—Zn—N2—C1	175.2 (2)	Zn—N1—C12—C10	175.37 (19)
O7—Zn—N2—C11	165.96 (15)	C8—N1—C12—C11	−179.6 (2)
O5—Zn—N2—C11	78.08 (16)	Zn—N1—C12—C11	−5.2 (2)
O1—Zn—N2—C11	−101.33 (16)	C6—C10—C12—N1	−2.2 (4)
N1—Zn—N2—C11	−3.46 (15)	C5—C10—C12—N1	178.1 (3)
C11—N2—C1—C2	−0.7 (4)	C6—C10—C12—C11	178.4 (3)
Zn—N2—C1—C2	−179.3 (2)	C5—C10—C12—C11	−1.3 (4)
N2—C1—C2—C3	−1.8 (5)	N2—C11—C12—N1	2.3 (3)
C1—C2—C3—C9	1.4 (5)	C9—C11—C12—N1	−178.2 (2)
C9—C4—C5—C10	0.7 (5)	N2—C11—C12—C10	−178.2 (2)
C10—C6—C7—C8	0.4 (5)	C9—C11—C12—C10	1.3 (3)

### Hydrogen-bond geometry (Å, °)

**Table:**

*D*—H···*A*	*D*—H	H···*A*	*D*···*A*	*D*—H···*A*
O5—H51···O2^i^	0.87	1.79	2.655 (2)	174
O5—H52···O4^ii^	0.87	1.91	2.775 (2)	172
O6—H61···O1^ii^	0.91	1.93	2.809 (2)	161
O6—H62···O3	0.87	1.86	2.688 (2)	158
O7—H71···O3^iii^	0.96	1.85	2.769 (3)	160
O7—H72···O4^i^	0.83	1.97	2.797 (2)	173
C1—H1···O3^iii^	0.93	2.51	3.416 (3)	165

Symmetry codes: (i) *x*−1, *y*, *z*; (ii) −*x*+1, *y*+1/2, −*z*+3/2; (iii) −*x*+1, *y*−1/2, −*z*+3/2.
